# The decay and consolidation of effector-independent motor memories

**DOI:** 10.1038/s41598-022-07032-7

**Published:** 2022-02-24

**Authors:** Shancheng Bao, Jinsung Wang, David L. Wright, John J. Buchanan, Yuming Lei

**Affiliations:** 1 Department of Health and Kinesiology, Texas A&M University, College Station, TX 77843 USA; 2 Department of Kinesiology, University of Wisconsin-Milwaukee, Milwaukee, WI 53151 USA

**Keywords:** Learning and memory, Consolidation

## Abstract

Learning a motor adaptation task produces intrinsically unstable or transient motor memories. Despite the presence of effector-independent motor memories following the learning of novel environmental dynamics, it remains largely unknown how those memory traces decay in different contexts and whether an “offline” consolidation period protects memories against decay. Here, we exploit inter-effector transfer to address these questions. We found that newly acquired motor memories formed with one effector could be partially retrieved by the untrained effector to enhance its performance when the decay occurred with the passage of time or “washout” trials on which error feedback was provided. The decay of motor memories was slower following “error-free” trials, on which errors were artificially clamped to zero or removed, compared with “washout” trials. However, effector-independent memory components were abolished following movements made in the absence of task errors, resulting in no transfer gains. The brain can stabilize motor memories during daytime wakefulness. We found that 6 h of wakeful resting increased the resistance of effector-independent memories to decay. Collectively, our results suggest that the decay of effector-independent motor memories is context-dependent, and offline processing preserves those memories against decay, leading to improvements of the subsequent inter-effector transfer.

## Introduction

Inter-effector transfer of motor learning refers to a process whereby initial training with one effector leads to subsequent performance gains with the opposite untrained effector. Substantial evidence has demonstrated the presence of inter-effector transfer of learning across multiple motor tasks in humans^1–6^ and non-human primates^7–9^, and indicated that specific neural networks provide the gateway for such transfer^10–13^. For example, neuroimaging and behavioral studies revealed that the parietal cortex and its connectivity with the motor and visual regions are engaged during inter-effector transfer^1,2^. Electrophysiological studies using paired-pulse brain stimulation indicated that interhemispheric plasticity mediates inter-effector transfer^10,14^. Elucidating the mechanisms underlying inter-effector transfer of motor learning not only provides substantial insights into neural communications between the hemispheres but also has important implications for clinical rehabilitation, sports, and tool use. For example, human lesion studies showed that initial training with the non-paretic effector is beneficial to motor skill acquisition of the paretic effector in stroke patients^15,16^. Resistance training with one effector results in strength gains with the untrained effector^3,17^. Non-human primate studies showed that learning a tool-use task over two weeks with one effector increases performance gains in the opposite effector^8^.

Motor adaptation tasks, such as adapting to visuomotor or mechanical perturbations during reaching movements, are widely used to study our ability to apply what has been learned with one effector to the other^1,2,19^. Evidence has shown that the memories developed through motor adaptation comprise effector-independent and effector-dependent components^20–23^. For example, the extent of transfer of motor memories between the effectors typically ranges from 10 to 60%^1,6,18,24–26^, suggesting that the memories acquired during motor adaptation with one effector are not sufficient to show superb performance from the beginning of a subsequence adaptation session with the untrained counterpart. The extent of inter-limb transfer, however, can increase substantially when the untrained effector is allowed to simply experience the task solution actively (over 90% transfer)^21^, or even passively (over 70% transfer)^27^, in advance. These findings suggest that typical motor adaptation results in motor memories that can transfer across different effectors (i.e., effector-independent) and those that cannot (i.e., effector-dependent). This idea is consistent with the findings that modulating the activation of the primary motor cortex (M1), the key site of use-dependent learning^28–31^, influences the retention of motor adaptation within the same effector^32,33^, but not the opposite effector^34^. In contrast, disruption of the left posterior parietal cortex (PPC) blocks inter-effector transfer of motor adaptation^18^, suggesting that effector-independent and effector-dependent motor memories are encoded at different neural circuits.

Generally, motor adaptation produces memories that are fragile and susceptible to interference^35–38^, which can later be forgotten or unlearned in different contexts. For example, Kitago and colleagues^39^ proposed that the decay of motor memories following sensorimotor adaptation could occur in four distinct contexts: (1) switching off visual perturbation (washout), (2) a period of inactivity (sitting-idle), (3) removal of visual feedback (no-feedback), and (4) eliminating ongoing movement errors (error-free). They reported that motor memories would be present in the form of improved relearning (e.g., savings) after the “washout” and “sitting-idle” unlearning conditions, while the “no-feedback” and “error-free” unlearning conditions resulted in the abolition of subsequent savings. Kitago and colleagues^39^ suggested that the decay of memories involves two distinct yet complementary processes: (1) one process in which the decay is an active process whereby the newly adapted behavior reverts to baseline habits and (2) one process in which the decay is a form of passive forgetting of an internal model. If the decay of motor memories is supported by two distinct processes, how are the effector-independent memory components affected by these processes? Do these decaying processes make different contributions to the patterns of inter-effector transfer? In Experiment 1 and 2, we employed a series of unlearning conditions (washout, sitting-idle, visual-clamp, force-clamp, and no visual feedback) to examine the decay of effector-independent motor memories engaged in five different contexts and probed their influences on the resultant inter-effector transfer. We hypothesized that the extent of inter-effector transfer is not fixed but rather depends on the context in which the decay of effector-independent memories occurs.

Fragile motor memory traces can be stabilized and protected against decay during an offline consolidation period^40–42^. There is longstanding evidence that different motor memories follow different consolidation processes^43^. For example, some memories are stabilized over a period of wakefulness, while other memories are only consolidated during sleep^44–47^. This occurs because different neural networks contribute to different consolidation processes. For example, neuroimaging studies show that separate neural networks contribute to the consolidation of motor sequence learning and motor adaptation^48^ and implicit and explicit memory consolidation engage dissociable functional networks^49^. Recent findings show that effector-independent and effector-dependent memories engage different networks^18^, but it remains unclear whether these two types of memory, measured by transfer test, are affected by different consolidation processes. In the first two experiments, we found a lack of inter-limb transfer following some forms of decaying conditions. These results might indicate that consolidation of effector-independent memories is disrupted when decaying conditions occur immediately after learning. Thus, in Experiment 3 we examined whether an offline consolidation period would protect effector-independent memories against decaying conditions employed In Experiment 1 and 2. Previous studies of motor adaptation have demonstrated that 6-h of wakeful resting is a critical amount of time necessary for the completeness of offline consolidation^50–52^, while consolidation processes might be incomplete for intervals under 6-h and susceptible to memory interference^53–55^. We hypothesized that a 6-h window of offline consolidation would preserve effector-independent motor memories against decaying processes, leading to a great extent of inter-effector transfer.

## Results

### Experiment 1: effector-independent memories can be retrieved when the decay occurs with the passage of time or “washout” trials

**Figure 1:**
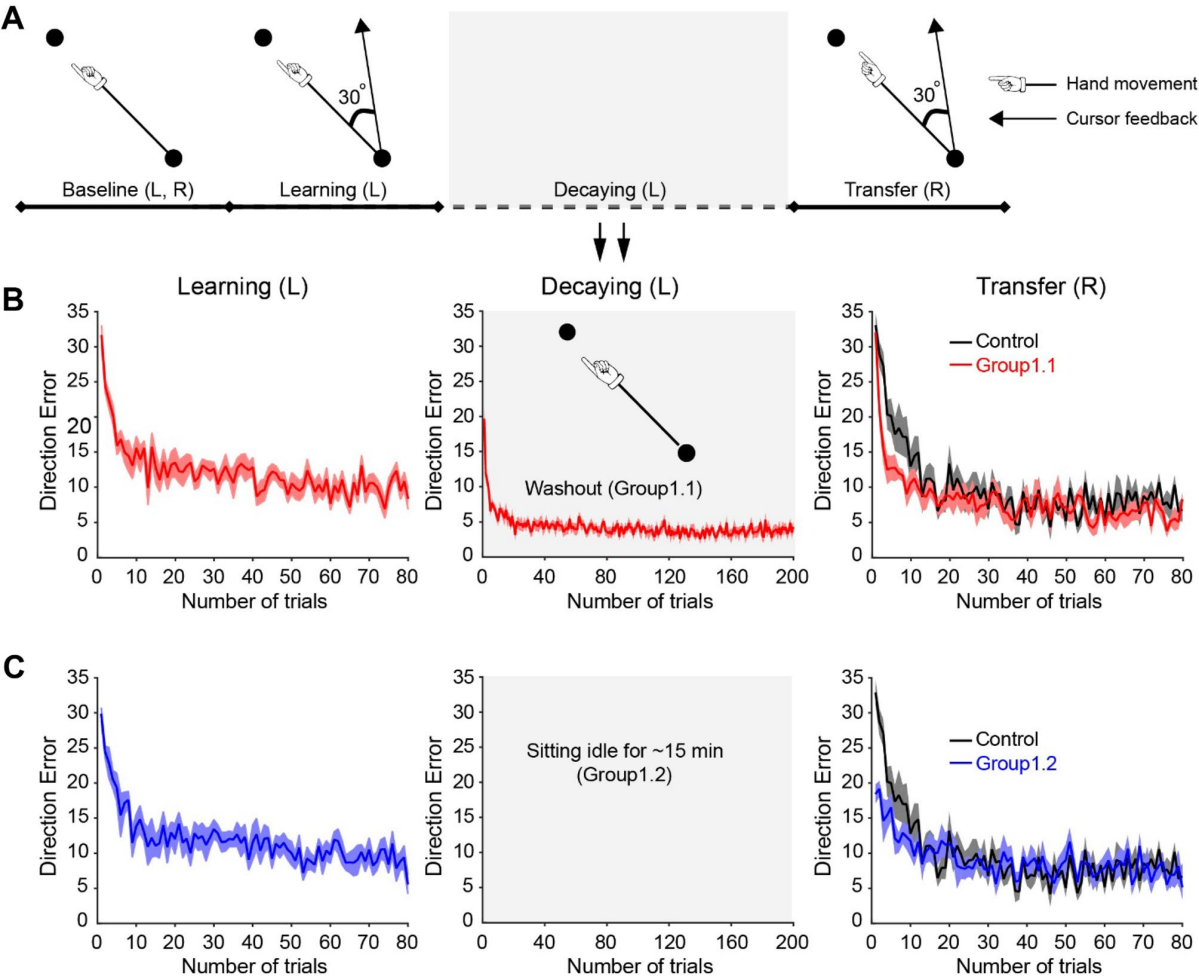
Protocols and the motor performance for Experiment 1. (**A**) The experimental protocol consisted of four sessions: baseline (veridical feedback), learning (30° CCW rotation), decaying, and transfer (30° CCW rotation with the opposite arm). The cursor feedback was CCW rotated by 30° from the hand-path in the learning and transfer sessions. (**B**) Mean time-course of the movement errors of the learning, decaying, and transfer sessions were plotted in red lines across trials for Group1.1. Standard errors were represented as the shaded area plot. Veridical cursor feedback was provided during the decaying session as shown in the mid panel. The control group’s right arm performance in the transfer session was plotted as black line in the right panel for comparison. (**C**) Mean time-course of the movement errors for Group1.2 in blue lines. The subjects sit idle during the decaying session (mid panel). The control group’s right arm performance was plotted in black in the right panel for comparison.

**Figure 2:**
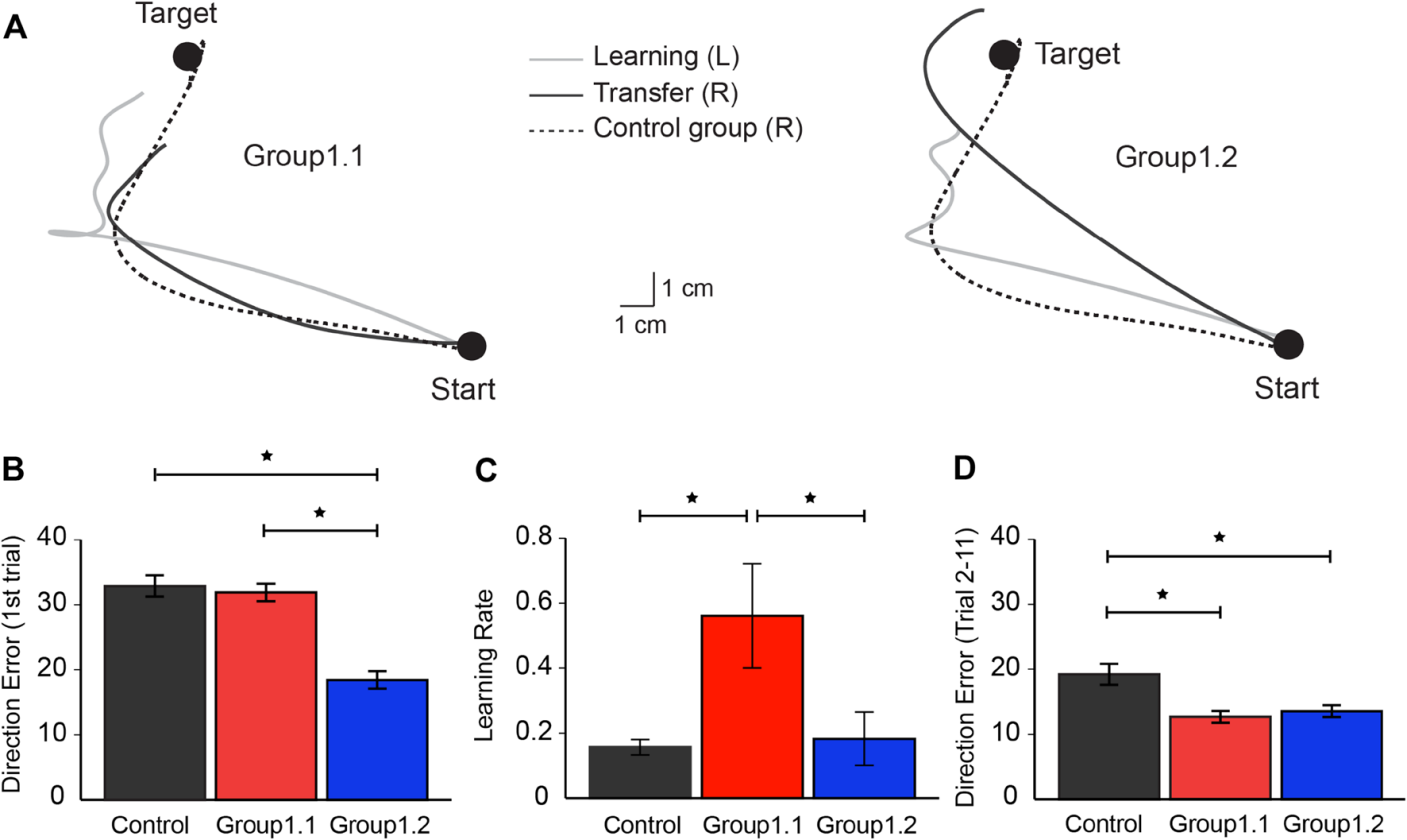
(**A**) Hand-paths from representative subjects observed on the first trials during the learning (gray) session and transfer (black) sessions for Group1.1 (left) and 1.2 (right). The hand-path for the control group was plotted in dashed line. (**B**) Mean errors of the first trial in the transfer session for Group1.1 (red), Group1.2 (blue), and control (black) (mean ± standard error). (**C**) Comparison of learning rates in the transfer session for the control and Group1.1 and Group1.2. (**D**) Comparison of savings in the transfer session using the alternative method. *p < 0.05.

During the learning session, all subjects made point-to-point movements with their left arm under conditions in which visual feedback was rotated 30° CCW (Fig. 1A). Figure 1B, C (left) depict changes in direction error (DE) across trials during the learning session for each group. As expected, the DEs were large due to the exposure to the novel visuomotor rotation, which decreased progressively throughout the session. A mixed ANOVA showed an effect of TRIAL (F_(2, 36)_ = 346.1, *p* < 0.001), but not GROUP (F_(1, 18)_ = 0.055, *p* = 0.8), or their interaction (F_(2, 36)_ = 0.867, *p* = 0.4) on DE. During the decaying session, Group1.1 experienced 200 “washout” trials. The DEs were returned to the baseline state by removing the visual rotation and providing error signals (asymptotic DE = 4.1 ± 1.6°, baseline DE = 5.0 ± 2.2°, *p* = 0.6; Fig. 1B, middle). Group1.2 sat idle for 840 s, which was the average amount of time taken by subjects in Group1.1 to complete 200 trials (Fig. 1C, middle). Figure 1B, C (right) show changes in DE across trials during the transfer session in Group1.1 and Group1.2 compared with a naïve control group in which subjects skipped the learning and decay sessions with the left arm but experienced the visual perturbation with the right arm for the first time. A mixed ANOVA showed an effect of TRIAL (F_(2, 54)_ = 359.0, *p* < 0.001), GROUP (F_(2, 27)_ = 20.0, *p* < 0.001), and their interaction (F_(4, 54)_ = 25.5, *p* < 0.001) on DE. This indicated that the changes in motor performance across trials were different among the three groups. Figure 2A illustrates the representative cursor-paths of a representative subject from Group1.1 and Group1.2 during the first trial of the learning and transfer session and from the control group during the first trial of the learning session. The cursor-paths (gray lines) all deviated largely from the target direction, indicating that the subjects were unable to compensate for the visual perturbation at the beginning of the learning session. The cursor-path (black line) during the transfer session from Goup1.1 was also largely biased towards the CCW direction, which indicated that the subjects in this group could not accurately move the cursor to the target during the initial period of inter-effector transfer. However, the cursor-path (black line) of the Goupr1.2 was more accurate compared with the control group (broken line), revealing that the adapted behavior could be retrieved by the untrained effector at the first trial of the transfer session. In agreement, Group data showed that DEs from the first trial during the transfer session were smaller in Group1.2 (18.4 ± 4.0°; Fig. 2B) compared with Group1.1 (31.9 ± 4.1°, *p* < 0.001) and the control group (32.8 ± 5.2°, *p* < 0.001). No difference was found between Group1.1 and the control group (*p* = 0.6). These results indicated that first-trial generalization existed after the passage of time, but was not present when decaying condition employed with “washout” trials on which the rotation was removed and error signals were provided. The learning rate was higher in Group1.1 (0.58 ± 0.4; Fig. 2C) compared with Group1.2 (0.15 ± 0.15, *p* = 0.01) and the control group (0.16 ± 0.08, *p* = 0.02), whereas no difference was observed between Group1.2 and the control group (*p* = 0.9). It is important to note that the calculated learning rate could be small if the performance exhibited immediate adaptation in the first trial upon perturbation exposure. Since Group1.2 exhibited immediate adaptation in the first trial during the transfer session, we estimated savings in an alternative way by calculating the mean error from the second to the 11th trial (Fig. 2D). Group1.1 and Group1.2 exhibited lower errors compared with the control group (*p* = 0.01 and 0.02, respectively), and no significant difference existed between Group1.1 and Group1.2 (*p* = 0.6). Taken together, these results indicated that the prior motor memories could be accessed by the untrained arm in Group1.1 and Group1.2.

### Experiment 2: effector-independent memories are abolished when the decay occurs in the absence of errors

**Figure 3:**
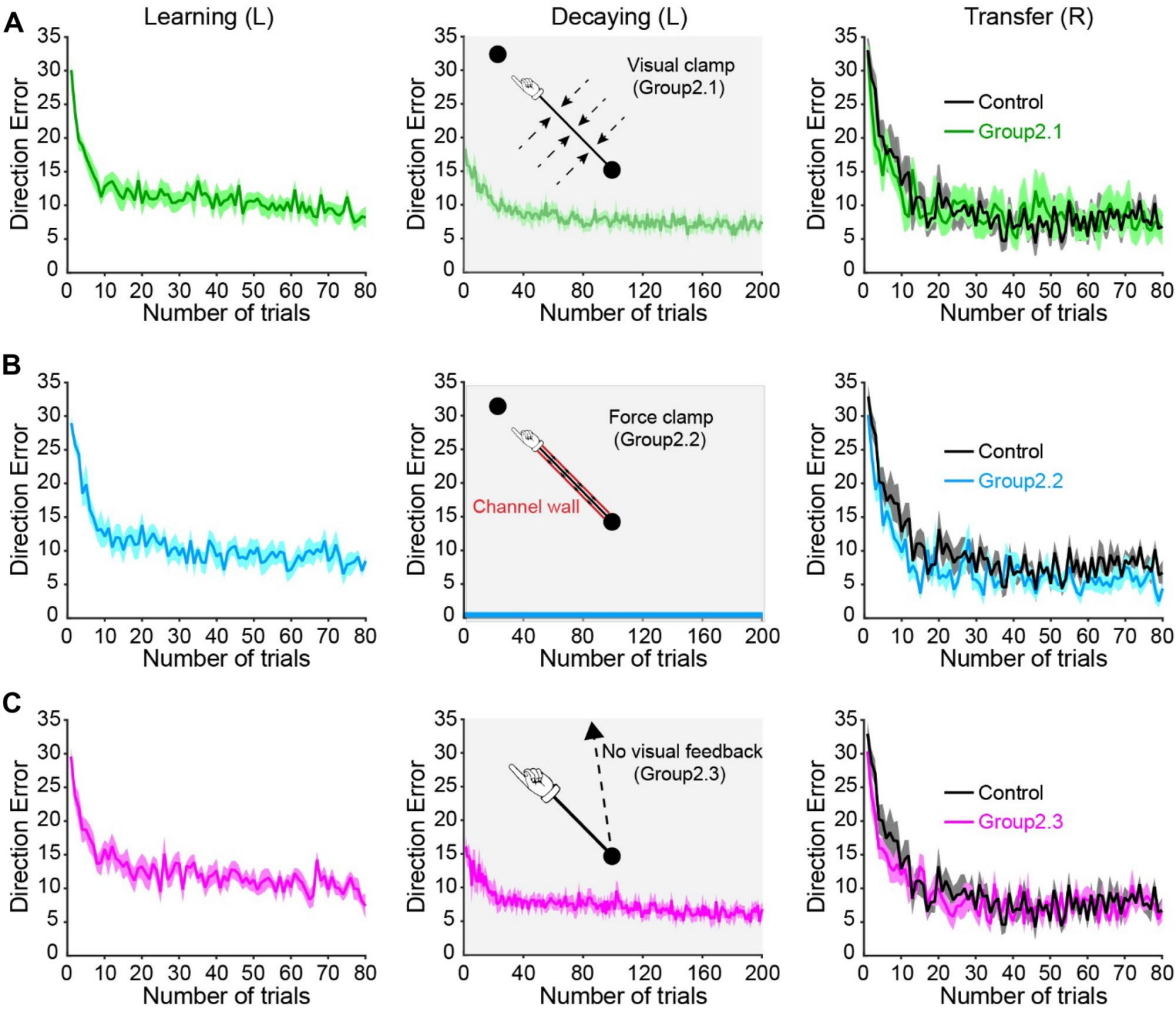
Performance for Experiment 2. (**A**) The movement errors of Group2.1 in the learning, decaying, and transfer sessions were plotted in green lines and shaded areas (mean ± standard error). During the decaying session, the cursor feedback was always on the straight line connecting the start and target circles. The black plot represented the control group’s performance in the transfer session. The performance of Group2.2 and 2.3 were depicted in (**B**) and (**C**), respectively. During the decaying session, a force channel restricted the hand-paths of Group2.2 along the straight line connecting the start and target circles, so the hand-paths were always straight and accurate. No cursor feedback was provided for Group2.3 during the decaying session, so the subjects reached towards the target based on their proprioception.

**Figure 4:**
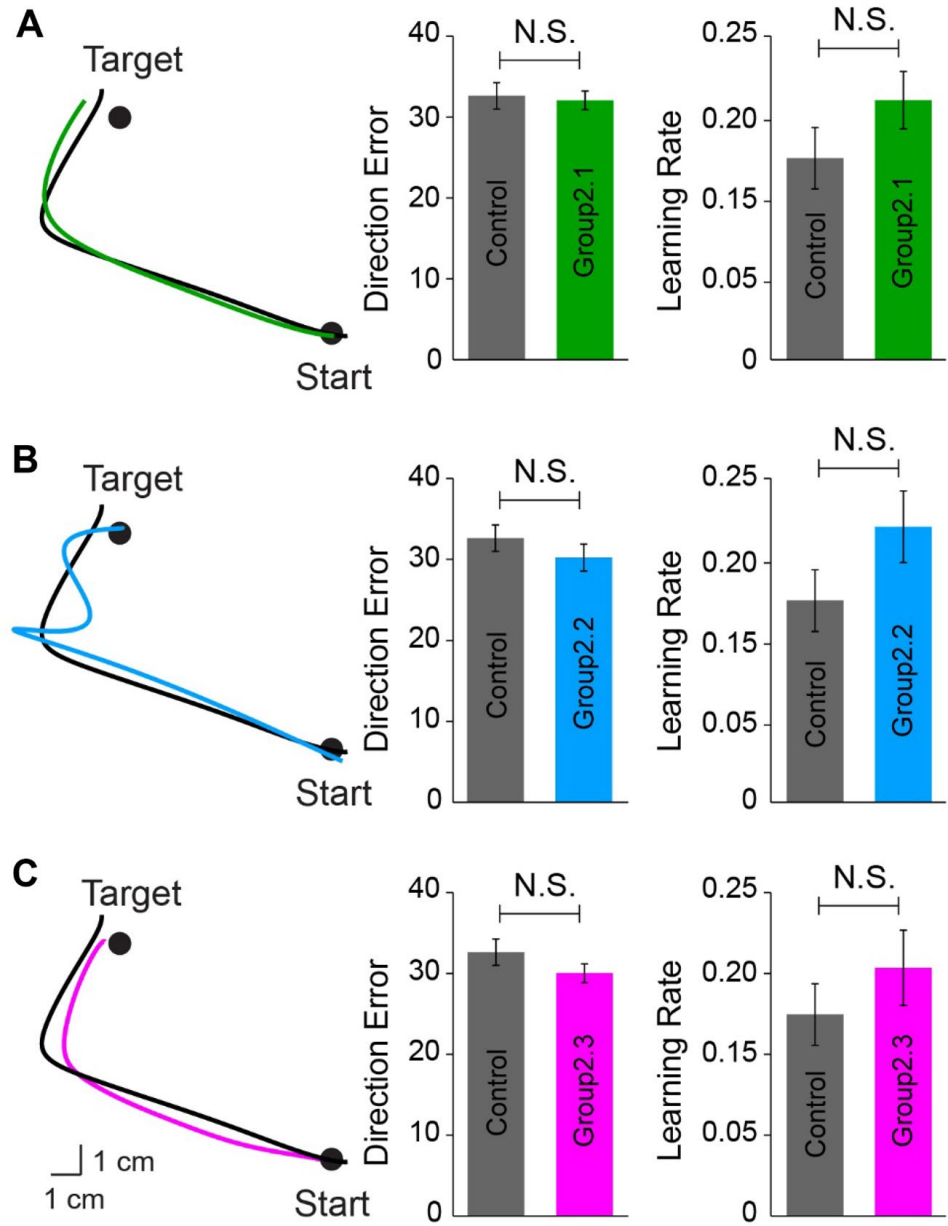
Hand-paths from representative subjects observed on the first trials during the transfer session were compared between the experimental groups (color) and the control (black) in the left panels for 2.1, 2.2, and 2.3, respectively (**A-C**, left). Mean errors of the first trial were plotted (mean ± standard error) in the mid panels (**A-C**, middle), and the learning rates were plotted on the right panels (**A-C**, right). N.S. indicates not significant (*p* > 0.05).

Motor adaptation is driven not only by sensory prediction errors (discrepancies between predicted and actual sensory consequences^56^), but also by task errors (discrepancies between predicted and actual task outcomes^57,58^). Motor memories are influenced in the absence of those errors that drive adaptation^37,39^. In Experiment 2, we examined the decay of effector-independent memories in the absence of errors using three different types of error-free trials. Figure 3A–C (left) depict changes in DE across trials during the learning session for three groups. A mixed ANOVA showed an effect of TRIAL (F_(2, 54)_ = 486.6, *p* < 0.001), but not GROUP (F_(2, 27)_ = 0.193, *p* = 0.8), or their interaction (F_(4, 54)_ = 0.123, *p* = 0.9) on DE. The DEs returned back toward baseline during the decaying sessions in Group2.1 (asymptotic DE = 6.1 ± 2.1°, baseline DE = 5.2 ± 2.6°, *p* = 0.4; Fig. 3A, middle) and Group2.3 (asymptotic DE = 7.1 ± 1.8°, baseline DE = 5.5 ± 2.6°, *p* = 0.3; Fig. 3C, middle). The DEs were set to be zero due to the force-clamp during the decaying session in Group2.2 (Fig. 3B, middle). Notably, the decay rates were slower during error-free trials on which error signals were clamped to zero (Group2.1 = 0.10 ± 0.08, *p* = 0.01) or removed (Group2.3 = 0.15 ± 0.13, *p* = 0.03) compared with “washout” trials (Group1.1 = 0.46 ± 0.36). Although the rate of decaying appeared faster in Group2.3 compared with Group2.1, this difference was not statistically significant (*p* = 0.3). Following the error-free trials, all subjects were re-exposed to the same perturbation for 80 trials with the opposite, untrained arm. A mixed ANOVA showed an effect of TRIAL (F_(2, 72)_ = 552.0, *p* < 0.001), but not GROUP (F_(3, 36)_ = 1.959, *p* = 0.2), or their interaction (F_(6, 72)_ = 0.63, *p* = 0.7) on DE during the transfer session (Fig. 3A–C, right). The lack of difference was further confirmed by calculating DEs at trial 1 during the transfer session, as well as the learning rate of the untrained effector. Figure 4A–C (left) show the representative movement trajectories of a representative subject from three error-free groups (colored line) and the control group (black line) upon initial exposure to the rotation during the transfer session. The trajectories were similarly deviated across all these subjects. DEs at trial 1 during the transfer session were not significantly different among the four groups (GROUP [F_(3, 39)_ = 0.813, *p* = 0.5]; Fig. 4A–C, middle). The ANOVA across the four groups using the learning rates showed no effect of GROUP (F_(3, 39)_ = 1.381, *p* = 0.3; Fig. 4A–C, right) either, indicating no savings following either error-clamp or no-error feedback trials. Overall, these results suggest that the effector-independent memories cannot be retrieved by the untrained effector when the decay of memories occurs in the absence of task errors.

### Experiment 3: offline processing consolidates effector-independent motor memories

**Figure 5:**
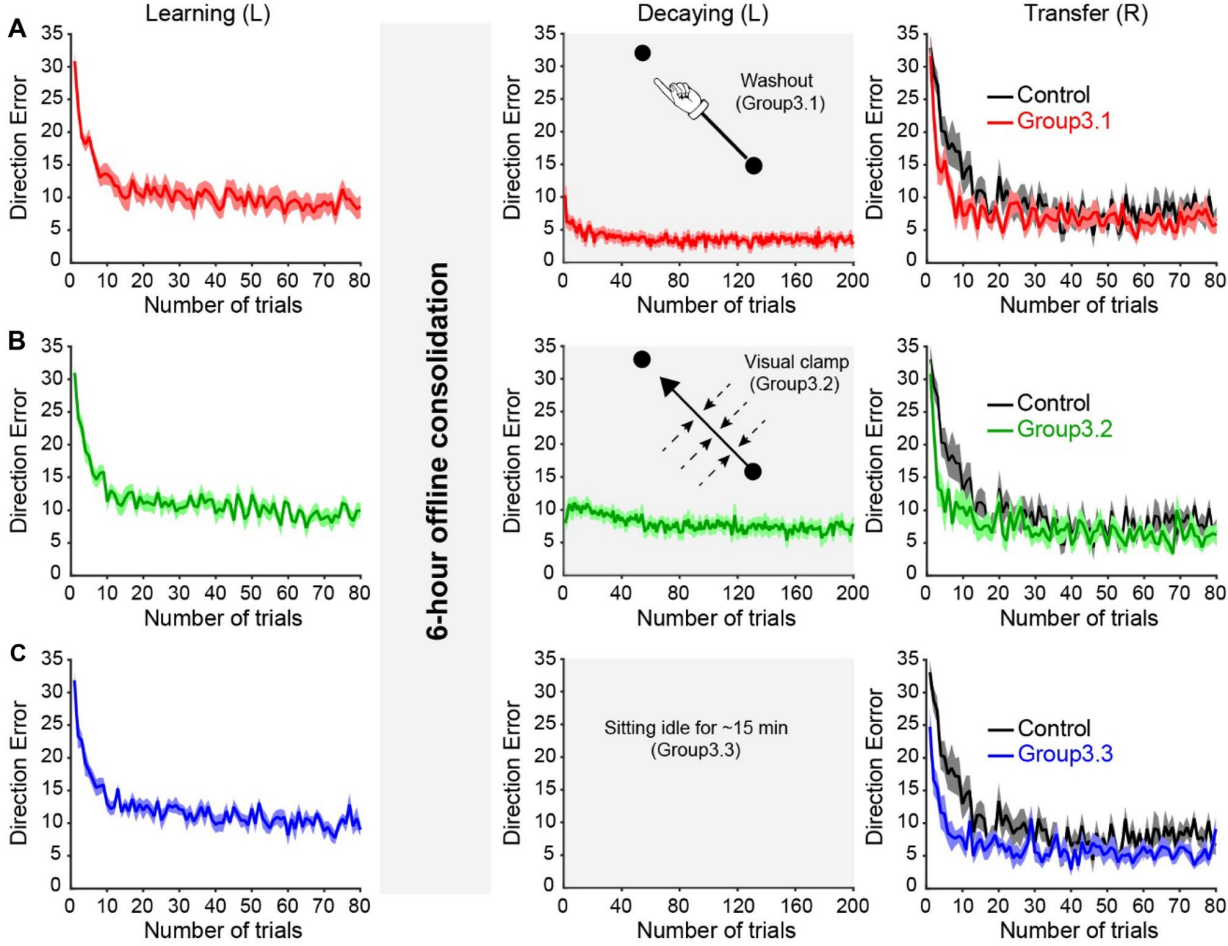
Performance of Experiment 3. (**A**) The movement errors of Group3.1 in the learning, decaying, and transfer sessions were plotted in red lines and shaded areas (mean ± standard error). The decaying session was 6 h after the learning, and the setup was identical to Group 1.1. The performance of the control group was plotted as the black line on the right panel. The movement errors of Group3.2 and 3.3 were plotted in (**B**) and (**C**), respectively. The setup of the decaying session for Group3.2 was identical to Group2.1, and the setup for Group3.3 was identical to that for Group1.2.

**Figure 6:**
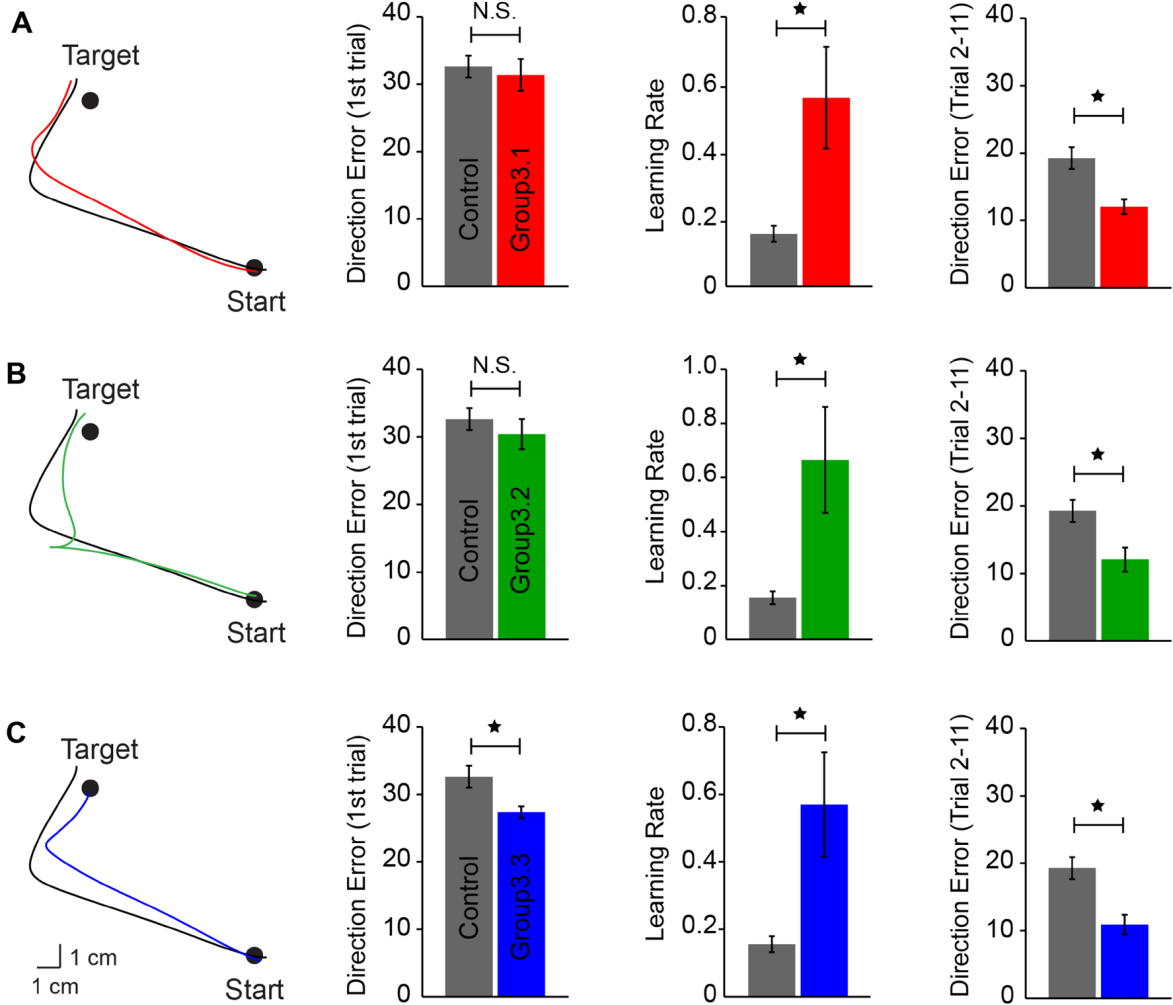
Hand-paths from representative subjects observed on the first trials during the transfer session were compared between the experimental groups (color) and the control (black) in the left panels for 3.1, 3.2, and 3.3, respectively (**A–C**, first column). Mean errors of the first trial were compared (mean ± standard error) in the second column panels (**A–C**,), the learning rates were plotted on the third column panels (**A–C**), and the mean errors from trial 2–11 were plotted on the fourth column panels (**A**–**C**). N.S. indicates not significant. *p < 0.05.

We next examined whether offline processing could consolidate effector-independent motor memories. Motor memories formed during adaptation can be stabilized over wakefulness^48,58^. Do effector-independent motor memories benefit from offline consolidation? To test that, we replicated the design of Experiment 1 and 2, except that participants underwent the learning session in the morning and experienced the transfer session 6 h later without any intervening sleep. Because we found no difference among the groups throughout the learning and transfer sessions in Experiment 2, we only replicated Group2.1 in which the subjects experienced visual error-clamp trials during the decaying session. Figure 5A–C (left) illustrate the performance during the learning session for each group. A mixed ANOVA showed an effect of TRIAL (F_(2, 54)_ = 511.5, *p* < 0.001), but not GROUP (F_(2, 27)_ = 0.63, *p* = 0.5), or their interaction (F_(4, 54)_ = 0.032, *p* = 0.9) on DE. Following 6 h of wakeful resting, the subjects were randomly assigned to one of three groups to participate in the decaying session. Group3.1 and Group 3.2 experienced 200 “washout” trials and error-clamp trials, respectively (Fig. 5A–B, middle). The DEs were returned to baseline in both groups (Group3.1: asymptotic DE = 3.7 ± 0.9°, baseline DE = 4.9 ± 1.4°, *p* = 0.2; Group3.2: asymptotic DE = 7.1 ± 1.9°, baseline DE = 5.3 ± 2.2°, *p* = 0.1). Group3.1 exhibited smaller DEs by the end of the decaying session compared with Group3.2 (*p* < 0.01). The subjects in Group3.3 sat idle for 860 s, which was the average amount of time taken by the subjects in Group3.1 and Group3.2 to complete the decaying session. During the transfer session, movement trajectories observed at trial 1 of the untrained effector was less deviated from the target line in Group3.3 (Fig. 6C, first column), whereas movement trajectories were noticeably more curved in Group3.1 and Group3.2 (Fig. 6A, B, first column). In agreement, average DEs from trial 1 during the transfer session were smaller in Group3.3 (27.6 ± 3.1°, *p* = 0.01; Fig. 6C, second column) compared with the control group (32.8 ± 5.2°). No difference in trial 1 DE was found between the other two groups (Group3.1 = 31.6 ± 7.1°, *p* = 0.7; Group3.2 = 30.6 ± 6.8°, *p* = 0.4) and the control group (Fig. 6A, B, second column). Effector-independent motor memories were present in the form of savings in all three group (Fig. 6A–C, third column). We found an effect of TRIAL (F_(2, 72)_ = 483.9, *p* < 0.001) and GROUP (F_(3, 36)_ = 7.411, *p* = 0.001), but not their interaction (F_(6, 72)_ = 1.68, *p* = 0.1) on DE. The one-way ANOVA showed an effect of GROUP (F_(3, 39)_ = 3.61, *p* = 0.02; Fig. 6A–C, third column) on learning rate. Post hoc analysis showed that learning rates increased significantly in all three experimental groups compared with the control group (Group3.1 = 0.56 ± 0.46, *p* = 0.02; Group3.2 = 0.67 ± 0.50, *p* = 0.01; Group3.3 = 0.57 ± 0.47, *p* = 0.02; Control group = 0.16 ± 0.08). Since Group3.3 exhibited smaller first-trial error, we also estimated the amount of savings in an alternative way by measuring the mean errors from the second to the 11th trial (Fig. 6A–C, fourth column). All the three groups (Group3.1, 3.2, and 3.3) showed lower errors than the control group (*p* = 0.02, *p* = 0.02, and *p* < 0.001, respectively), and there was no significant difference among the three groups (*p* > 0.5). Hence, 6-h offline consolidation was sufficient to protect effector-independent memories against decay across all movement contexts, even tested in the absence of errors. In addition, we conducted a mixed ANOVA to examine the effect of 6-h offline consolidation on the preservation of motor memories by comparing the data among Experiment 1 (Group1.1 and Group1.2), Experiment 2 (Group2.1), and Experiment 3 (Group3.1, Group3.2, and Group3.3). We found an effect of GROUP on learning rate during the transfer session. Group3.2 and Group3.3 showed significantly higher learning rate compared with Group2.1 (*p* = 0.02) and Group1.2 (*p* = 0.03), respectively. No significant difference was observed between Group1.1 and Group3.1 (*p* = 0.9), indicating that motor memories were present in the form of savings in Group3.1 since faster learning was observed in Group1.1 and Group3.1. Altogether, these results supported the view that effector-independent motor memories were preserved following 6-h offline consolidation.

## Discussion

In the present study, we provide clear evidence that the decay of effector-independent motor memories is highly context-dependent. Specifically, effector-independent motor memories can be recalled by the untrained effector when motor memory decay occurs with the passage of time (sitting-idle) or at “washout” trials on which movements are made in the same context as the baseline. The retrieval of effector-independent memories is blocked if the decay occurs when movements are made in novel contexts (i.e., reaching without visual feedback, reaching in the error clamp). The brain can protect and stabilize the motor memories during daytime wakefulness or sleep^44,59,60^. We further suggest that 6 h of wakeful resting following motor memory formation can protect effector-independent memories against decay to a certain extent, resulting in a great extent of inter-effector transfer.

### The decay of effector-independent motor memories

Motor adaptation with one effector develops motor memories that comprise effector-independent components, which can subsequently be accessed by its counterpart^1,22,61^. However, those memories formed during the adaptation are inherently transient^35–37^, and can be forgotten or unlearned immediately and automatically^39^. Here, we demonstrated that the manner in which the decay of motor memories occurs with one effector has significant consequences for the subsequent performance with the other effector. We found substantial initial transfer from the trained to the untrained effector when the decay occurred with the passage of time, indicating that effector-independent motor memories did not decay away following a short period of inactivity and were still available to benefit subsequent performances of the untrained effector. These data are congruent with previous findings showing that individuals can recall the motor memory of adapted behavior after an idle period^39,54,62,63^. When motor memory decay occurred at “washout” trials, memories were retrieved with the untrained effector in the form of savings. We suggested that internal memory models of the novel sensorimotor conditions were unlearned following the “washout” trials. This is supported by our results that the errors returned to baseline during the decaying session, and that the errors in the first trial of the transfer session were similar to those in the first trial in the control group. However, motor memories formed through sensorimotor adaptation were preserved because no novel contexts were involved during the “washout” trials; instead, the brain just switched between the perturbation context and the baseline context. During the transfer session, the brain seems to use the first trial to probe whether the memories formed with the trained effector were useful for subsequent performances with the untrained effector^21,22,64^. If the motor memories were determined useful after the first trial, the brain would utilize them to facilitate subsequence performances, thus resulting in savings. This interpretation is in agreement with a computational study of motor adaptation showing that the learner is able to rapidly switch between adapted behavior and baseline habits after “washout” trials, resulting in savings^62^.

In Experiment 2, we found the lack of first-trial generalization and savings when motor memory decay occurred during error-free trials on which errors were artificially clamped to zero (visual- and force-clamp) or removed (no-feedback), indicating that the motor memories developed through motor adaptation were inhibited following these three conditions and could not be retrieved by the untrained effector. These results suggested that motor memories were influenced when movements were made in new contexts in which the tasks exhibited different properties. Indeed, the subjects were requested to perform movements in new or unfamiliar contexts (i.e., reaching without visual feedback, reaching in the error clamp), which they have not experienced previously (or would not perform in typical contexts). In this case, the motor memories associated with the novel visuomotor rotation (from the learning session) and the motor memories associated with the new contexts (from the decaying session) would compete for the same neural resources, especially given that the same motor effector was used in both sessions. In consequence, the prior motor memories would be overwritten by the newly formed motor memories. This argument is in agreement with the previous findings that multiple motor memories stored in the brain can compete with each other for retrieval^22,53,64,66,67^.

### The consolidation of effector-independent motor memories

When errors were absent during the decaying session, effector-independent motor memories were abolished. We argued that motor memories, which did not have enough time to be consolidated yet, were susceptible to interference if the brain actively engaged in a new movement context. If true, then offline consolidation occurring over a period of time should be sufficient for preserving those memories against decay and interference. We tested this in Experiment 3 by providing a 6-h window of wakeful resting between the learning and decaying sessions. Previous studies of motor adaptation have shown that a 6-h period is a critical amount of time necessary for offline consolidation^51,52,54^, and sleep does not add any additional benefits to offline processing^36,48,51,58^. In Experiment 3, we found that prior motor memories could be accessed by the untrained effector in the form of savings in all three groups (Group3.1, 3.2, and 3.3). We suggest that the inter-effector transfer in the form of savings occurs because training with one effector produces a memory of errors, which is stabilized by offline consolidation in Group3.1. This memory of errors is retrieved by the untrained effector, which increases error sensitivity to produce savings when reencountering familiar errors. This interpretation is in agreement with one influential theory proposed by Herzfeld and colleagues^68^, who suggested that savings results from the memory of errors that increase sensitivity to the experienced errors. This view is supported by the absence of savings in Group2.1, 2.2, 2.3, and the published study^57^, when the previous correction of task errors is absent**.**

We examined the decay of effector-independent motor memories by expanding the experimental design presented by Kitago and colleagues^36^, who suggested that the decay of memories might be due to forgetting of an internal model and a model-free phenomenon. Numerous studies have previously demonstrated that motor adaptation involves model-free learning components, including reinforcement learning^39,56,69,70^ and use-dependent plasticity^22,23,27,71^. Reinforcement theory suggests that if specific actions are associated with successful outputs and these actions will be recalled under the same condition^56,69^. Use-dependent plasticity represents the mechanism that repetitive practice of the same movement will shape the reorganization of neural circuits in the motor cortex, which enables fast and automatized retrieval of the same performance in subsequent trials. Our previous studies suggest that use-dependent learning involves neural presentations that are specifically tied to a particular effector^6,27,64,72^. Thus, motor memories acquired via use-dependent learning with one effector cannot be retrieved by the untrained effector, which explains the phenomenon of limited inter-effector transfer after an offline consolidation process.

### Functional considerations

A critical question that we attempted to address in the current study was how distinct decaying mechanisms would mediate the nature of effector-independent motor memories associated with motor adaptation and whether effector-independent memories could be consolidated against these decaying processes. Here, we demonstrated that context-dependent decay is a feature of effector-independent motor memories, and those memories can be protected and stabilized by offline consolidation. Our finding has an important implication for movement rehabilitation for neurological disorders or musculoskeletal injuries. For example, initial training with the non-paretic hand can lead to subsequent performance gains in the paretic hand in stroke patients^15^. As our finding indicates, however, the beneficial effect of the non-paretic hand training in rehabilitation could be eliminated if newly acquired motor memories formed with the non-paretic hand are engaged in different contexts without memory consolidation. Some strategies might be available to minimize such decaying effects. For example, exercise^73,74^ and non-invasive motor cortex stimulation^23,66,75,76^ can cause motor memories to be consolidated faster. Whether and how such methodologies can minimize the effect of distinct decaying environments (thus to maximize the effect of non-paretic side training) remains to be investigated.

## Materials and Methods

### Subjects

Ninety healthy right-handed individuals (18–35 years old, 61 females) with no history of neurological or musculoskeletal disorder participated in this study. All subjects were naïve to our apparatus, the paradigm, and the purpose of the study. All experimental protocols were approved by the Institutional Review Board of Texas A&M University. All subjects gave written informed consent prior to participation, which was approved by the local ethics committee at Texas A&M University in accordance with the Declaration of Helsinki.

### Apparatus

A bilateral robotic exoskeleton KINARM (BKIN Technologies Ltd, Kingston, ON, Canada) was used to collect movement data. The setup of the robotic system has been described in our previous paper^21–24^. The subjects were seated on the height-adjustable chair with both arms supported horizontally by the KINAM exoskeleton. The linkages of the exoskeleton were adjusted to custom-fit each subject according to their arm length and geometry^6,21–24^. The exoskeleton was incorporated with a virtual reality system that projected visual targets on a horizontal display to make them appear in the same plane as the arm^21–24,27^. Direct vision of the arm was blocked; a white cursor representing the location of the index fingertip was provided to guide the subject’s reaching movement. The 2-D position of the index fingertip was sampled at 1000 Hz, low-pass filtered at 15 Hz, and differentiated to yield resultant movement velocity^23^. Data were processed and analyzed offline using MATLAB (Mathworks, Natick, MA).

### Experimental design

The general motor task was to control the white cursor to perform reaching movements from a start circle to a target circle (1 cm in radius white circles, 10 cm apart). The target circle was located in the 45° direction from the start circle. The subjects first moved their index finger (cursor) to the start circle, and stopped there. A trial was initiated and the start circle turned green when the cursor remained within the start circle at a speed < 0.1 cm/s. Then, the subjects were instructed to move the cursor from the start circle to the target rapidly and accurately once the target appeared. Movements were required to have a reaction time (RT) of < 600 ms and a movement time (MT) within 400–800 ms. If RT > 600 ms, no visual feedback regarding movement trajectory was available and the trial was aborted. The color of the target turned red or blue if the movement was too fast (MT < 400 ms) or too slow (MT > 800 ms), and such trials were aborted as well. The target color turned green when the performance met the MT requirement (400 ms < MT < 800 ms).

Our aims were (1) to understand the decay of effector-independent motor memories engaged in different contexts, and (2) to probe their influences on a resultant inter-effector transfer task. In a series of tasks, we varied contexts and examined how those memory traces decayed across contexts (Experiment 1 and 2). This was followed up with another series of tasks (Experiment 3) in which the hypothesis that offline consolidation preserves effector-independent memories against decay across all contexts was tested. Three groups (*n* = 30) of subjects participated in Experiment 1. Group1.1 (*n* = 10) and Group1.2 (*n* = 10) experienced four sessions (Fig. 1A): baseline, learning, decaying, and transfer. In the baseline session, the subjects repeated the reaching movements for 80 trials (40 trials with each arm, counterbalanced) with veridical visual feedback to be familiarized with the general motor task. In the learning session, all subjects adapted with the left arm to a visual display that was rotated 30° counterclockwise (CCW) about the start circle for 80 trials. In the decaying session, Group1.1 experienced 200 “washout” trials with the left arm on which the visual perturbation was removed, and error feedback was provided throughout the entire reaching movement in each trial. Group1.2 sat idle for about 15 min, which was around the same amount of time that Group1.1 spent to complete the decaying session. Group1.1 and Group1.2 were re-exposed to the same visual perturbation to perform reaching movements with the right arm for 80 trials in the transfer session. The subjects were neither informed about the existence of visuomotor rotation throughout the experiment, nor were clear about whether the rotation would be on or off in the coming trials. A group of subjects (*n* = 10) only experienced baseline (40 trials with each arm, counterbalanced) and transfer (80 trials with right arm) sessions (learning and decaying sessions skipped). This control group served as our benchmark for right arm learning performance without any transfer gains from learning with the left arm, and their performance was also analyzed and compared in Experiment 2 and 3.

In Experiment 2, we used different types of error-free trials to examine the potential effect of context on the decay of effector-independent memories. Three groups (*n* = 30) of subjects participated in Experiment 2. All groups experienced four sessions (baseline, learning, decaying, and transfer sessions), in which the baseline, learning, and transfer sessions were identical to the reaching task in Group1.1. In the decaying session, Group2.1 (*n* = 10) experienced 200 visual error-clamp trials in which the visual feedback of movement trajectory was clamped to a straight line between the start and target circle and thus spatially independent of actual performance. Group2.2 (*n* = 10) experienced 200 force-clamp trials in which the subjects moved in a force channel, bounding the straight-line path between the start and target circle by generating reactive forces with a stiffness of 1500 N/m and damping of 5 N/m/s^77^. The channel was implemented as in the equation below:

$$\begin{aligned} \left[ {\begin{array}{*{20}c} {{\text{F}}_{x} } \\ {F_{y} } \\ \end{array} } \right] & = \left[ {\begin{array}{*{20}c} { - 1061} & { - 1061} \\ { - 1061} & { - 1061} \\ \end{array} } \right]{ }\left[ {\begin{array}{*{20}c} {\text{X}} \\ Y \\ \end{array} } \right] + \left[ {\begin{array}{*{20}c} { - 3.54} & 0 \\ 0 & { - 3.54} \\ \end{array} } \right]{ }\left[ {\begin{array}{*{20}c} {{\text{V}}_{x} + {\text{V}}_{y} } \\ {{\text{V}}_{x} + {\text{V}}_{y} } \\ \end{array} } \right] + {\text{THETA}}\;\left( {\text{N}} \right) \\ {\text{THETA}} & = \left\{ {\begin{array}{*{20}c} {\left[ {\begin{array}{*{20}c} {37} \\ {37} \\ \end{array} } \right],\quad X + Y > 0.0354} \\ { \left[ {\begin{array}{*{20}c} {1061} & {1061} \\ {1061} & {1061} \\ \end{array} } \right] \left[ {\begin{array}{*{20}c} {\text{X}} \\ Y \\ \end{array} } \right],\quad \left| {X + Y} \right| \le 0.0354} \\ {\left[ {\begin{array}{*{20}c} { - 37} \\ { - 37} \\ \end{array} } \right],\quad X + Y < - 0.0354} \\ \end{array} } \right. \\ \end{aligned}$$

where F_x_ and F_y_ (N) were forces applied along the medial–lateral and anterior–posterior directions, X and Y (m) were the spatial positions obtained by the relative displacement from the start circle, V_x_ and V_y_ (m/s) were the x and y components of the end-point velocity. A velocity-dependent viscous force was added to maintain stability in the direction that was perpendicular to the channel. Note that sensory prediction error was clamped to zero in the visual- and force-clamp trials. Group2.3 experienced 200 trials with no visual feedback of performance errors. As similar to Experiment 1, the subjects were not informed about the nature of visuomotor perturbation, and were not clear when the perturbation was turned on or off.

Offline processing transforms a newly acquired motor memory from a fragile state to a stable state^48,54,61^. In Experiment 3, we examined the effect of 6 h of wakeful resting between the learning and decaying session on the consolidation of effector-independent motor memories. Three groups (*n* = 30) of subjects participated in Experiment 3. All subjects experienced baseline and learning sessions in the morning. After 6 h, the subjects were randomly assigned to three experimental groups to participate in the decaying session. For Group3.1, the decaying session had 200 “washout” trials. For Group3.2, the decaying session had 200 error-clamp trials. Subjects in Group3.3 sat idle for about 15 min, which was the same amount of time that Group3.1 and Group3.2 spent to complete the decaying session. Following the decaying session, all subjects were exposed to the same visual perturbation with the untrained effector for 80 trials.

### Data analysis

To examine performance accuracy, we measured the direction error (DE), which was the angular difference between a vector from the start circle to the target and another vector from the hand position at movement start to that at peak arm velocity. To assess the extent of inter-effector transfer, we compared the DE of the first trial in the transfer session to that observed on the first naïve learning trial in the control group who learned with the right arm without any transfer gains from learning with the left arm. DE at trial 1 (first-trial generalization) was used because subsequent trials in the transfer session were not a “pure” measure of generalization, confounded by the learning with the untrained effector. In addition, we also calculated the learning rate to determine if the rate of motor adaptation with the right arm following initial training with the left arm was different across the groups. The learning rate was obtained by fitting with a single decaying exponential function for each subject based on the following equation: ${\text{DE}}\left( n \right) = {\text{A}}*{\text{exp}}\left( { - {\text{R}}*n} \right) + {\text{B}} \cdot {\text{R}}$ was the individual learning rate, A and B were two constants, and n was the trial index. An Increased learning rate in the experimental group compared with the control group indicated that training with the left arm resulted in improved performance in the right arm in the form of savings. It is important to note that the rate constants could be small if the performance exhibited immediate adaptation in the first trial upon perturbation exposure^57,78,79^. Therefore, savings was also measured by calculating the mean value of first few trials (except for trial 1) if immediate adaptation was observed in the first trial. Specifically, the trial difference tended to approach zero by the 12th trial in this study, so savings was estimated by measuring the mean errors from the second to the 11th trial.

All statistical analyses were conducted using MATLAB and SPSS (version 27; IBM). Normal distribution was tested by the Shapiro–Wilk's test. When normal distribution could not be assumed, data were log transformed. Mixed ANOVAs were performed to determine the effect of GROUP and TRIAL [initial bias (trial 1), early learning (mean of trial 2–10), and asymptote learning (mean of trial 61–80)] on DE. One-way ANOVAs and independent t-tests were conducted to determine if the DEs and learning rates were different across the groups in each experiment. Tukey post hoc analysis was used to test for significant comparisons. Alpha value was set at 0.05. Group data were presented as mean ± standard error.
